# A biomarker of opioid-induced respiratory toxicity in experimental studies

**DOI:** 10.1016/j.isci.2023.106520

**Published:** 2023-03-30

**Authors:** Marieke Hellinga, Marijke Hyke Algera, Rutger van der Schrier, Elise Sarton, Monique van Velzen, Albert Dahan, Erik Olofsen, Marieke Niesters

**Affiliations:** 1 Department of Anesthesiology, Leiden University Medical Center, 2333 ZA Leiden, the Netherlands; 2 PainLess Foundation, 2333 ZA Leiden, the Netherlands

**Keywords:** Health sciences, Pharmacology, Toxicology

## Abstract

Opioids are commonly used painkillers and drugs of abuse and have serious toxic effects including potentially lethal respiratory depression. It remains unknown which respiratory parameter is the most sensitive biomarker of opioid-induced respiratory depression (OIRD). To evaluate this issue, we studied 24 volunteers and measured resting ventilation, resting end-tidal PCO_2_ (P_ET_CO_2_) and the hypercapnic ventilatory response (HCVR) before and at 1-h intervals following intake of the opioid tapentadol. Pharmacokinetic/pharmacodynamic analyses that included CO_2_ kinetics were applied to model the responses with focus on resting variables obtained without added CO_2_, HCVR slope and ventilation at an extrapolated P_ET_CO_2_ of 55 mmHg (${\dot{V}}_{E}$55). The HCVR, particularly ${\dot{V}}_{E}$55 followed by slope, was most sensitive in terms of potency; resting variables were least sensitive and responded slower to the opioid. Using ${\dot{V}}_{E}$55 as biomarker in quantitative studies on OIRD allows standardized comparison among opioids in the assessment of their safety.

## Highlights

• Opioids are drugs of abuse and produce potentially lethal respiratory depression (RD)
• Isohypercapnic ventilation is the most sensitive biomarker of opioid-induced RD
• Baseline variables are least sensitive biomarkers of opioid respiratory depression
• Comparative and experimental opioid studies should use isohypercapnic ventilation

## Graphical abstract

**Figure:**
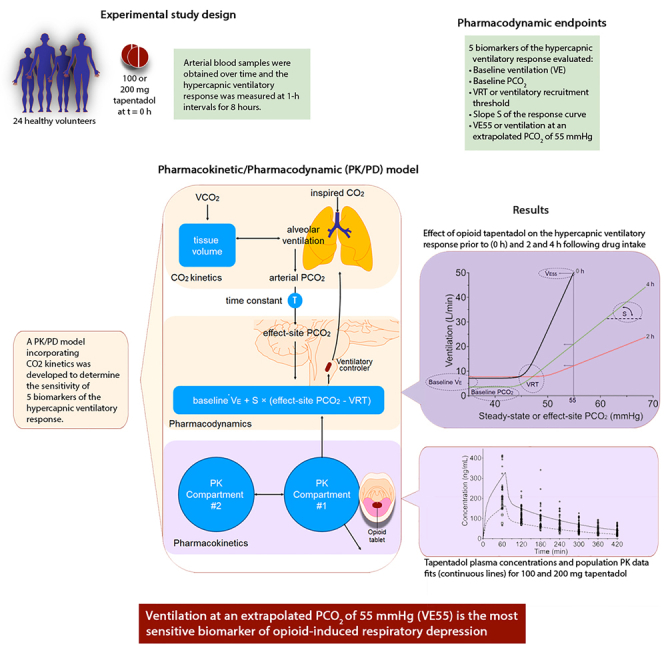

## Introduction

**Figure 1:**
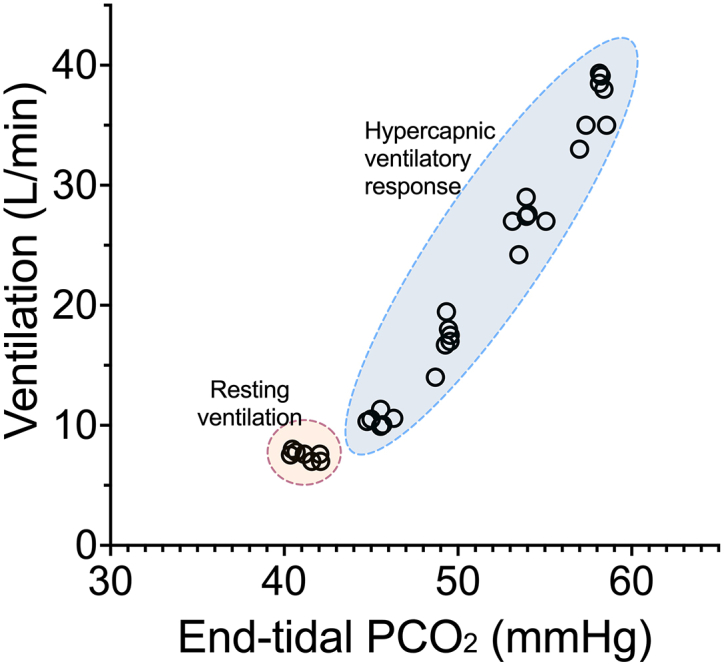
The hypercapnic ventilatory response (HCVR) The response was obtained by performing steps in end-tidal PCO_2_. Each circle is a 1-min ventilation average. The orange cloud includes data obtained without any added inspired CO_2_ (resting or poikilocapnic data). The blue cloud includes data obtained at inspired CO_2_ concentrations and is the HCVR.

Opioids are commonly used in clinical practice to manage acute and chronic pain and suppress the sympathetic stress response during surgery.^1^ However, opioids are toxic and possess several adverse effects including abuse, addiction and potentially life-threatening respiratory depression (RD). The combination of addiction and RD is the root cause of the current opioid crisis and numerous deaths in the US and other Western nations.^2^^,^^3^^,^^4^ Opioids are effective and possibly even the most effective pain killers available.^1^ Consequently and despite their known toxicity, their clinical use will continue and new opioids are being developed with the promise of reduced respiratory toxicity.^5^^,^^6^ To validate whether opioids have an improved respiratory safety profile compared to other drugs of the same class, a comprehensive understanding of their respiratory effects on the human ventilatory control system is necessary. A large number of studies explored the effects of opioids on breathing,^1^ but it remains unclear which of the many respiratory variables is the most sensitive and consequently most useful biomarker of the impact that opioids have on ventilatory control. In the current study, we address this issue by performing a physiology based pharmacokinetic/pharmacodynamic (PK/PD) analysis of the effect of an opioid on the hypercapnic ventilatory response (HCVR), integrating “resting” data, obtained before CO_2_ inhalation (Figure 1), in the analysis. The model incorporates the CO_2_ alveolar mass balance,^7^^,^^8^^,^^9^ and describes the horizontal part of the curve where ventilation is independent of CO_2_.^10^ The resultant is a function of ventilation versus steady-state PCO_2_. Four biomarkers were extracted from the analysis: resting ventilation (${\dot{V}}_{E}$), resting end-tidal PCO_2_ (P_ET_CO_2_), the ventilatory recruitment threshold (VRT or the end-tidal PCO_2_ at which ventilation begins to rise on CO_2_ inhalation) and the slope of the HCVR. Furthermore, we calculated ventilation at an extrapolated end-tidal PCO_2_ of 55 mmHg (${\dot{V}}_{E}$55).^11^^,^^12^ These five biomarkers were subsequently modeled over time using a sigmoid E_MAX_ model (with parameters potency, C_50_, and hysteresis half-life, h½) to determine which one was most sensitive to the opioid. Experiments were performed in healthy and opioid-naïve male and female volunteers, who either received a low dose or a high dose of the opioid tapentadol.

## Results

**Figure 2:**
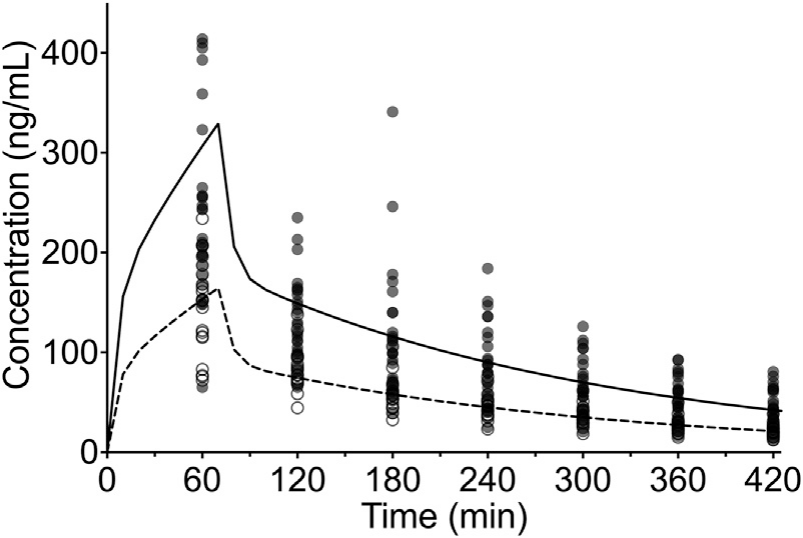
Plasma concentrations of the opioid tapentadol of all participants Open circles are the low dose (oral 100 mg), gray circles the high dose (oral 200 mg). The lines through the data are the population fits (broken lines, 100 mg tapentadol; continuous line, 200 mg tapentadol).

Individual plasma concentrations and population data fits following low- and high-dose tapentadol ingestion are given in Figure 2. The PK data were analyzed with a two-compartment model, with the following parameter estimates: Volume of compartment 1 (V_1_) = 90 ± 6 (median ± standard error of the estimate) L/70 kg, volume of compartment 2 (V_2_) = 557 ± 24 L/70 kg, elimination clearance from compartment 1 (CL) = 197 L/h (for a 70 kg individual) with between-subject variance 0.06 ± 0.02, intercompartmental clearance (CL_2_) 726 ± 34 L/h, tapentadol absorption time from intake to blood 1.2 ± 0.1 h and residual error 0.015 ± 0.002.

**Figure 3:**
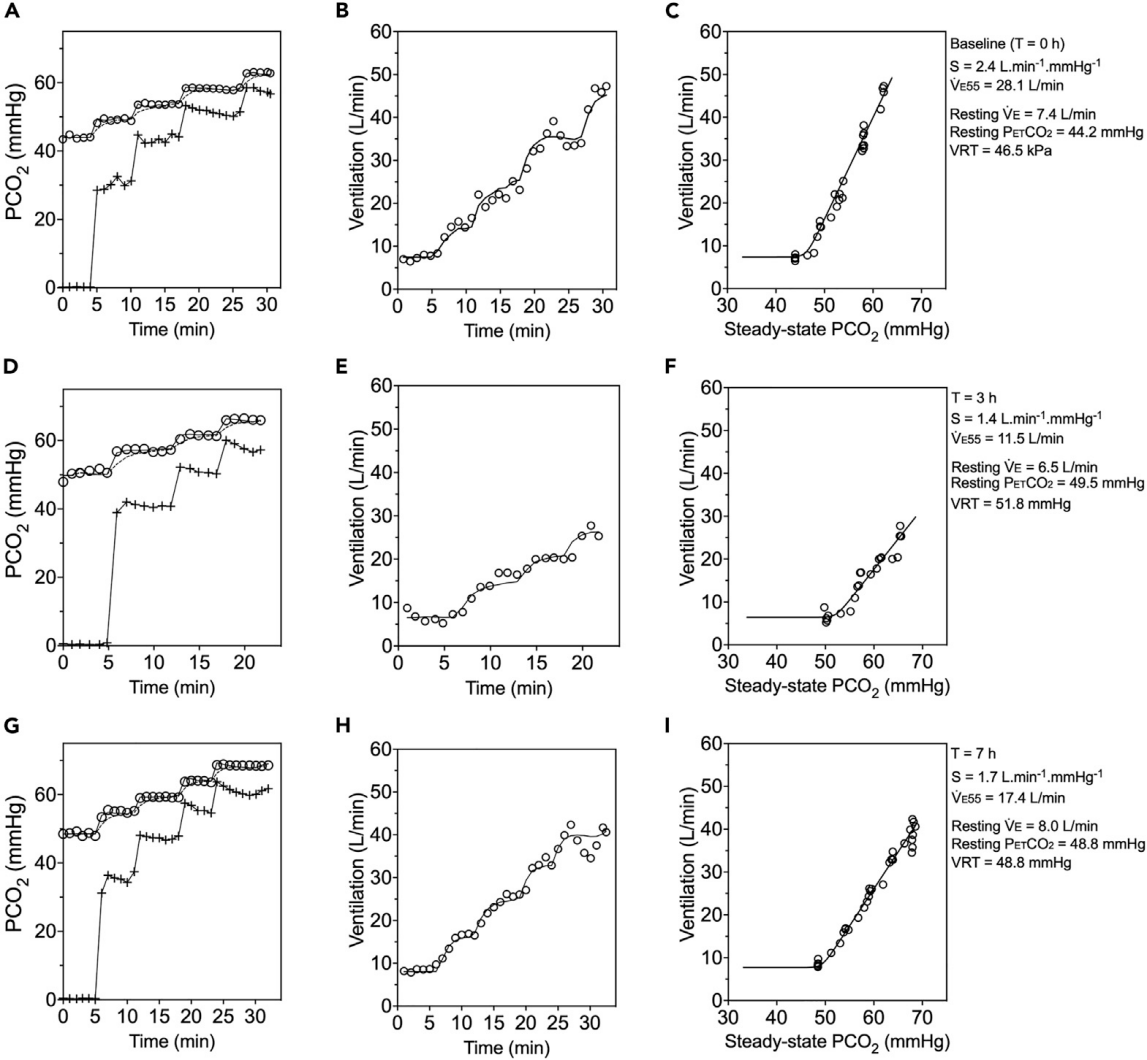
Development of the HCVR and effect of the opioid on the different components of the hypercapnic ventilatory response Data are from a single subject (#005). The symbols are the measured data, the lines the estimated model output. (A–C) analysis of the PCO_2_ and ventilation data before any drug administration, (D–F) and (G–I) analysis of data obtained 3 and 7 h after 200 mg tapentadol intake, respectively. Panels (A, D, and G) depict the transition from measured inspired PCO_2_ (+) to measured end-tidal PCO_2_ (circles); line through the end-tidal data is the estimated end-tidal PCO_2_; the broken line through the data is the effect-site or brain tissue PCO_2_. Panels B, E and H depict the ventilation data fits (circles are ventilation data, the line through the data the model fit). Panels (C, F, and I) depict the HCVR (continuous line) as function of effect-site PCO_2_. The circles are measured data. Parameter estimates are given for each time point with S the slope of the hypercapnic ventilator response, ${\dot{V}}_{E}$55 extrapolated ventilation at a PCO_2_ of 55 mmHg 0 and VRT the ventilatory recruitment threshold.

**Figure 4:**
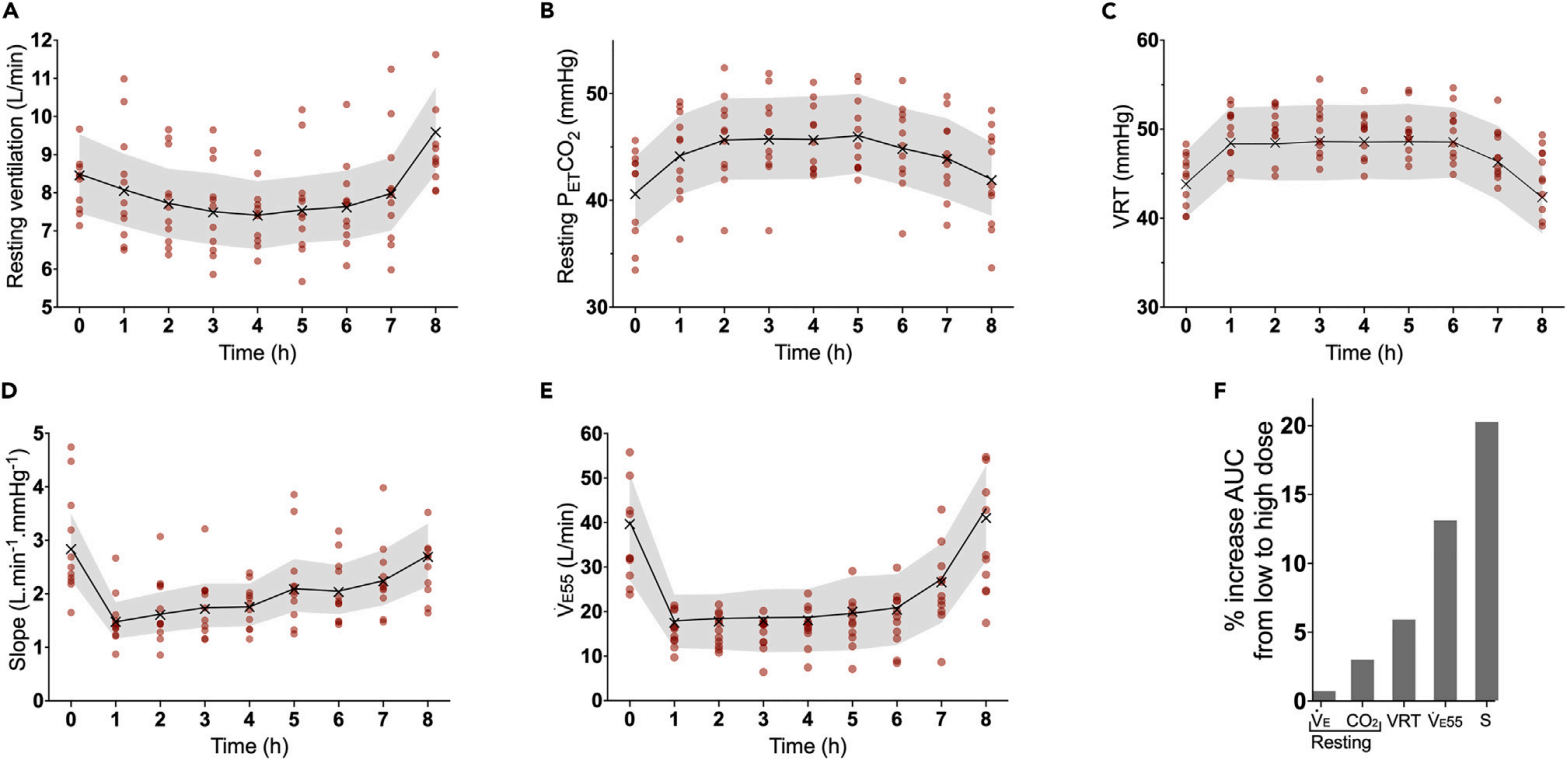
Pharmacodynamic analysis of the effect of 200 mg tapentadol on the base indices of the HCVR over the 8-h course of the experiment (A) Resting ventilation (ventilation before any added inspired CO_2_). (B) Resting end-tidal PCO_2_. (C) The ventilatory recruitment threshold (VRT). (D) The slope of the HCVR (S). (E) Ventilation at an extrapolated PCO_2_ of 55 mmHg (${\dot{V}}_{E}$55). (F) The percentage change in parameter estimates between 100 and 200 mg oral tapentadol. The 100 mg data are not depicted. Red circles are individual parameter estimates over time, × the median values of the posterior distribution from the NONMEM Bayesian step ±2.5 and 97.5 percentiles (gray area) and estimated population fits (continuous lines). All presented data are derived from the modeling analysis.

**Figure 5:**
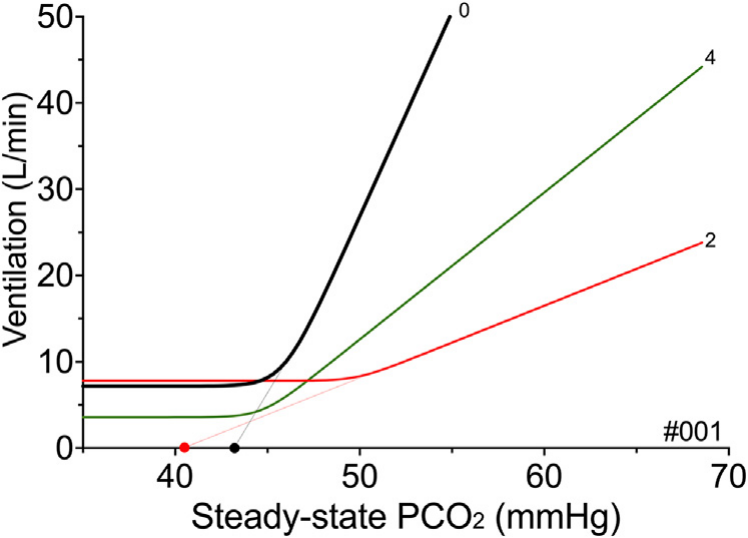
Effect of the opioid tapentadol on the hypercapnic ventilatory response Differential effect of resting data and data obtained at an elevated PCO_2_ observed in subject 001 and derived from the data modeling. The black line is the HCVR curve obtain before any opioid intake (time 0). The horizontal part of the curve is the so-called dog-leg and reflects resting data without added CO_2_ (poikilocapnic data). The line deflects upwards at the ventilatory recruitment threshold and turns into the response obtained at added inspired CO_2_ (isohypercapnic data). The next response, red line, is obtained 2 h after 200 mg oral tapentadol intake. The slope of the HCVR has decreased to its lowest value measured, whereas the resting ventilation has not changed from control, indicative of the greater sensitivity of the response obtained at added inspired CO_2_ than resting data obtained at poikilocapnia. At t = 4 h after opioid intake, green line, the slope returns toward control, value, whereas resting ventilation now shows its peak depression effect, indicative of the slower response of resting data compared to data obtained at isohypercapnia. On the xaxis, the so-called apneic thresholds (or PCO_2_ at apnea) for the control curve and the one obtained at t = 2 h are depicted as black and red symbols, respectively. The rising part of the curve is commonly analyzed by the function Ventilation = S × (end-tidal PCO_2_ – B) where B is the apneic threshold.

Examples from one subject of the pharmacodynamic analyses are given in Figure 3 (the symbols are the measured data, the lines the estimated model output). It shows the analytical steps from inspired PCO_2_ to the HCVR with its parameters resting ${\dot{V}}_{E}$, resting P_ET_CO_2_, VRT and the slope of the HCVR. Note that the HCVR is plotted versus the steady-state CO_2_ partial pressure. In our model the steady-state PCO_2_ is equivalent to the effect-site PCO_2_, i.e., the site where CO_2_ is presumably sensed (see below). The extracted parameter estimates and calculated ${\dot{V}}_{E}$55 were next analyzed using sigmoid E_MAX_ models. The individual parameter estimates over time, the median values of the posterior distribution from the NONMEM Bayesian step ±2.5 and 97.5 percentiles and estimated population fits are given in Figure 4 for the high-dose opioid dataset (panels A-E; all data presented are derived from the modeling analysis). These pharmacodynamic parameter estimates are given in Table 1. For all parameters, shape factor γ was not different from 1 and therefore fixed to 1. Resting ${\dot{V}}_{E}$, resting P_ET_CO_2_ and VRT had similar dynamics with similar values for potency (C_50_ = 0.81 ng/mL, meaning that at this concentration their value drops by 50%) and hysteresis (h½ = 1.3 h), whereas the dynamics of parameters S and ${\dot{V}}_{E}$55 were distinct from the other parameters (p < 0.01; Table 1). The picture that emerges from the analyses is that resting ${\dot{V}}_{E}$, resting P_ET_CO_2_ and VRT respond slower with lesser sensitivity to the opioid than S and ${\dot{V}}_{E}$55. Comparing S and ${\dot{V}}_{E}$55, ${\dot{V}}_{E}$55 was most sensitive in terms of potency with a C_50_ value of 0.08 ng/mL, i.e., 10-fold more sensitive than resting parameters, versus C_50_ for S of 0.23 ng/mL. S displayed a lesser hysteresis (h½ = 0.6 versus 0.9 h). The differences in dynamics between the HCVR, particularly S and resting parameters is exemplified in Figure 5, showing the effect of 200 mg tapentadol at t = 0 (control) and 2 and 4 h after opioid intake. The decrease in slope in this specific subject peaked at 2 h (and afterward S returned toward pre-drug values), whereas resting parameters had their peak at t = 4 h.

**Table 1:** Pharmacodynamic parameter estimates

	Estimate± SEE	Inter-subject variability (ω^2^)± SEE (%CV)
Resting ${\dot{V}}_{E}$ (L/min) at t = 0 h	8.3 ± 0.2	0.017 ± 0.006 (13)
Resting PCO_2_ (mmHg) at t = 0 h	41 ± 0.8	0.007 ± 0.003 (8)
VRT at t = 0 h (mmHg)	44 ± 0.8	0.005 ± 0.003 (7)
S at t = 0 h (L·min^−1^·mmHg^−1^)	2.5 ± 0.2	0.134 ± 0.047 (38)
${\dot{V}}_{E}$55 at t = 0 h (L/min)	37.1 ± 2.8	0.121 ± 0.113 (35)
C_50_ resting ${\dot{V}}_{E}$ (ng/mL)	0.81 ± 0.08	0.066 ± 0.020 (26)
C_50_ resting PCO_2_ (ng/mL)	0.81 ± 0.08	0.066 ± 0.020 (26)
C_50_ VRT (ng/mL)	0.81 ± 0.08	0.066 ± 0.020 (26)
h½ resting ${\dot{V}}_{E}$ (h)	1.3 ± 0.1	–
h½ resting PCO_2_ (h)	1.3 ± 0.1	–
h½ VRT (h)	1.3 ± 0.1	–
C_50_ S (ng/mL)	0.23 ± 0.09	0.589 ± 0.343 (33)
h½ S (h)	0.6 ± 0.3	–
C_50_${\dot{V}}_{E}$55 (ng/mL)	0.08 ± 0.01	0.105 ± 0.042 (33)
h½ ${\dot{V}}_{E}$55 (h)	0.9 ± 0.1	–
		**Within-subject variability (σ)± SEE**
resting ${\dot{V}}_{E}$ (L/min)		0.44 ± 0.03
resting PCO_2_ (mmHg)		1.50 ± 0.09
VRT (mmHg)		1.90 ± 0.10
S (L·min^−1^·mmHg^−1^)		0.49 ± 0.03
${\dot{V}}_{E}$55 (L/min)		3.46 ± 0.22

Resting indicates data obtained with inspired CO_2_; t = 0 h indicates parameter values prior to any tapentadol administration; VRT is the carbon dioxide partial pressure at the ventilatory recruitment threshold; S the slope of the HCVR; h½ the hysteresis parameter (blood-to-effect-site equilibration half-life); C_50_ is the concentration at steady-state causing 50% effect; SEE is the standard error of the estimate; ω^2^ is the variance for inter-subject variability with %CV the coefficient of variation calculated as $\sqrt{\exp\left(\omega^{2}\right)-1}\times 100$, where. Within-subject variability (σ) is calculated as the $\sqrt{\sigma^{2}}$ where $\sigma^{2}$ is the variance for inter-subject variability, with its SEE given as [(SEE of σ^2^/σ^2^)/2] ×$\sqrt{\sigma^{2}}$.

When comparing the opioid doses, the dose-response effect was largest for parameter S, whereas the smallest dose effect was observed for resting ${\dot{V}}_{E}$ (in comparison, dose effect S > ${\dot{V}}_{E}$55> VRT > resting P_ET_CO_2_> resting ${\dot{V}}_{E}$; Figure 4F). This indicates that parameters derived from the HCVR were more sensitive to an increasing opioid dose compared to resting data.

## Discussion

It is important to evaluate and compare the respiratory effects of existing opioids and those in development to ensure their safe clinical use. There are different biomarkers of the respiratory effect of opioids, but their sensitivity and utility remain understudied. To quantify the effect of opioids on ventilatory control, some studies analyzed the effect of opioids on resting respiratory variables (*e.g.* respiratory rate) and estimated rather high C_50_ values. This implies that the tested opioids had a low sensitivity or potency for RD and consequently are erroneously considered relatively safe. For example, Mildh et al.^13^ studied the effect of two potent intravenous opioids, alfentanil and fentanyl, in eight healthy volunteers, and measured plasma concentration, resting ventilation, arterial PCO_2_ and respiratory rate under poikilocapnic conditions, i.e., without strictly controlled end-tidal CO_2_. PK/PD modeling was used to estimate the opioid concentration that produces 50% decrease in resting ${\dot{V}}_{E}$, respiratory rate and increase in PCO_2_. The C_50_-values were 195 ng/mL for alfentanil and 3.5 ng/mL for fentanyl, with identical values for the three distinct biomarkers. We earlier estimated C_50_-values obtained for isohypercapnic ${\dot{V}}_{E}$ (i.e., ${\dot{V}}_{E}$ measured at a clamped end-tidal PCO_2_ of 50–55 mmHg) in the order of 1.0 ng/mL for fentanyl and 50 ng/mL for alfentanil.^14^^,^^15^ This indicates a 3-fold lower opioid sensitivity in data obtained at poikilocapnia compared to data obtained at isohypercapnia. The reason for the differences is that at poikilocapnia the opioid-induced reduction in ${\dot{V}}_{E}$ and subsequent rise in arterial PCO_2_ interact within the CO_2_ chemoreflex feedback loop in such a way that the net changes in either parameter are minimized.^16^ One might argue that the data obtained under resting or poikilocapnic conditions are more realistic, however, we would counter-argue that the model estimates depend on many experimental conditions that require consideration, such as the dose and the speed at which the opioid is administered. We showed that an opioid that is administered rapidly, causing a high peak in plasma and brain concentration, rapidly silences respiratory rhythm generation, whereas the same drug that is administered slowly allows accumulation of CO_2_ that will offset (part of) the RD.^8^ Modeling such effects without considering CO_2_ kinetics will then result in a high C_50_ value that holds the assumption of relative safety. Hence, we consider the C_50_-estimates derived from the opioid effect on isohypercapnic ventilation a more realistic approximation of the opioid’s respiratory potency, i.e., not underestimating the opioid’s potency. The current results provide additional arguments for this opinion.

We compared the pharmacodynamic properties of five biomarkers of ventilation, of which two were obtained before any CO_2_ inhalation, resting ${\dot{V}}_{E}$ and P_ET_CO_2_, and three indices were part of the HCVR curve, VRT, S and ${\dot{V}}_{E}$55. Resting data together with VRT behaved similarly in terms of the opioid pharmacodynamics with similar values for C_50_ and h½. S and ${\dot{V}}_{E}$55 had distinct pharmacodynamics with some differences in their parameter estimates. Differences between the two sets of parameters were that resting data and VRT displayed a lesser potency and had a slower onset/offset in opioid effect than S and ${\dot{V}}_{E}$55. The potency ratios were such that S and ${\dot{V}}_{E}$55 were 3.5 and 10 times more potent than the other parameter set, whereas h½ differed by a factor of 2 with a more rapid onset/offset for S and ${\dot{V}}_{E}$55. We relate the slower response and lesser opioid sensitivity of the two resting parameters and VRT to the interactive chemoreflex-related effect of arterial CO_2_ on ${\dot{V}}_{E}$ and vice versa*,* as discussed above.^16^ The higher sensitivity of ${\dot{V}}_{E}$55 relative to S, may be related to the fact that ${\dot{V}}_{E}$55 is dependent on the slope as well as on the position of the HCVR curve (Figure 5). The high opioid sensitivity observed for ${\dot{V}}_{E}$55 indicates that this specific index is the most sensitive and, as we argue, useful biomarker when quantifying the impact of opioids on ventilatory control. The choice of “best” clinical opioid when comparing these drugs for safety during pain relief, however, is determined by their utility function or therapeutic ratio.^14^^,^^17^ In other words, opioids that have the largest separation between C_50_ for analgesia and RD, under the condition that C_50_ for analgesia < C_50_ for RD, might be considered the opioid with theoretically a greater likelihood for analgesia than RD, although in the clinical setting this still depends on dose and speed of drug administration. We here define C_50_ for analgesia as a 50% increase in ability to fend nociceptive stimuli (*e.g.* a 50% increase in pain pressure threshold) and C_50_ for RD as a 50% depression of isohypercapnic ventilation or ${\dot{V}}_{E}$55. Unfortunately, most opioids show greater or equal potency for RD than analgesia. For example, for oxycodone, C_50_s are 0.08 ng/mL for analgesia and 0.05 ng/mL for RD (unpublished observation), for fentanyl equivalent values are 1.8 and 1.0 ng/mL,^14^ whereas for morphine C_50_ was equal for both endpoints (10 ng/mL).^18^ In all cases, the analysis of RD was based on isohypercapnic breathing (P_ET_CO_2_ fixed at 50–55 mmHg) and the analysis of analgesia was based on experimental studies on antinociception. Hence, one cannot blindly extrapolate these data to the clinical setting. Our analysis is particularly useful in experimental comparative studies of opioids and other drugs that affect ventilatory control, though.

We administered tapentadol as example opioid. In contrast to classical opioids, it has a dual mechanism of action.^19^ It activates the μ-opioid receptor and simultaneously inhibits neuronal reuptake of noradrenaline. This later mechanism is analgesic as noradrenaline inhibits post-synaptic nociceptive neurons in the spinal cord that express α_2_-adrenergic receptors. Because activation of the adrenergic system has little respiratory effects, we expect that tapentadol has a respiratory advantage over other opioids. However, the noradrenergic effect takes time to develop, very similar to the time it takes for selective antidepressants with the same mechanism of action to produce effective mood improvement (selective serotonin and noradrenaline reuptake inhibitors such as venlafaxine and duloxetine). Further studies in patients taking tapentadol for at least 2 weeks are needed to determine whether the separation of C_50_ for analgesia and RD exceed that of, for example oxycodone.

### Limitations of the study

Traditionally, the HCVR is analyzed by fitting the ventilation data to the following formula ${\dot{V}}_{E}$ = S × (P_ET_CO_2_ – B),^16^ where B is the extrapolated P_ET_CO_2_ at zero ventilation (apneic threshold) and S equals per definition the combined sensitivity of the peripheral and central chemoreceptors. We plotted the apneic thresholds of a control HCVR curve and a curve at peak RD, as based on ${\dot{V}}_{E}$55, in Figure 5. When considering the magnitude of the opioid effect, the leftward shift of the apneic thresholds gives little information without any knowledge on the change in slope. Biomarker ${\dot{V}}_{E}$55 captures both and hence is our choice rather than just S or B for quantifying drug effect on ventilatory control.

The HCVR is a function of PCO_2_ (Figure 1). In steady-state hypercapnic experiments, in which inspired CO_2_ is raised and P_ET_CO_2_ and ${\dot{V}}_{E}$ are at steady state, i.e., both variables show no further increase over time, we assume that P_ET_CO_2_ closely approaches brain tissue or effect-site PCO_2_. Under these circumstances, the HCVR is measured at the site of the central chemoreceptors, assuming that the ventilatory response to CO_2_ arises predominantly from the activation of central chemoreceptors. We do stress that the effect-site PCO_2_ does not correspond to the anatomical sites of CO_2_ sensing. The sites of central CO_2_ sensing are still under debate and the mechanism of CO_2_ sensing depends on complex mechanisms not captured in our pharmacodynamic model. Steady-state conditions are not met when non-steady-state experiments are conducted or experiments in which CO_2_ rises without any steady state in P_ET_CO_2_ or ${\dot{V}}_{E}$.^20^ The resultant HCVR is then not a function of the effect-site or steady-state PCO_2_. However, if the analysis considers CO_2_ kinetics, the response will be a function of the effect-site or steady-state PCO_2_ irrespective of the method of inducing hypercapnia. Our analysis is therefore a major advantage over other methods as it does not require an experimental steady state in ${\dot{V}}_{E}$ or P_ET_CO_2_.

We modeled ${\dot{V}}_{E}$ as linear function of PCO_2_ beyond the VRT (Figures 3 and 5). Others used a non-linear exponential function to describe the ${\dot{V}}_{E}$-PCO_2_ relationship and included resting data.^7^^,^^9^ It is our consistent observation that the HCVR over the PCO_2_ range measured (beyond the VRT) is best approximated by a linear function (Figures 1, 3, and 5). Moreover, the dynamics of the initial horizontal part of the curve is dissimilar compared to the dynamics of the rest of the curve, as we demonstrated here, and therefore requires a distinct approach. We are aware that at higher PCO_2_ values the curve flattens and the complete HCVR has a sigmoid shape. We argue that our approach best reflects the physiological changes that occur following inhalation of up to 9% CO_2_.^21^

### Conclusions

We systematically analyzed the effect of a potent opioid on five biomarkers of the HCVR and observed that ${\dot{V}}_{E}$55, calculated from the HCVR curve, is the most sensitive quantitative index of opioid-induced RD. In addition, we showed that resting variables such as resting ${\dot{V}}_{E}$ and P_ET_CO_2_ respond slower and with a lesser sensitivity to the opioid than the slope of the HCVR and ${\dot{V}}_{E}$55 and hence underestimate the opioid respiratory effect. We argue that ${\dot{V}}_{E}$55 is the appropriate index to be used in experimental studies on the effect of opioids (and other drugs) on ventilatory control. Adoption of this index as biomarker of opioid effect on ventilation will reduce the rather diverse outcome of opioid respiratory studies and will allow a clear quantitative comparison among opioids in the assessment of their safety.

## STAR★Methods

### Key resources table

**Table:** 

REAGENT or RESOURCE	SOURCE	IDENTIFIER
**Software**		

NONMEM (software package for non-linear mixed effects modelling)	Icon Development Solutions, Ellicott City, MD, USA.^9^	version VII
GraphPad Prism v9.5.1 for MacOS	https://www.graphpad.com	N/A

**Other**		

Tapentadol immediate release tablets 100 and 200 mg	Grünenthal GmbH, Aachen, Germany	N/A

### Resource availability

#### Lead contact

Further information and requests for resources should be directed to the corresponding author/lead contact Prof. dr. Albert Dahan (a.dahan@lumc.nl).

#### Materials availability

This study did not generate new unique reagents.

### Experimental model and subject details

#### Study conduct and ethics

The current report is part of a larger project in which two opioids (oxycodone and tapentadol) were tested; here we present the results of the study on tapentadol. The study was performed from January 2019 to December 2020 after approval of the study was obtained from the Medical Research Review Board Leiden-the Hague-Delft (METC-LDD, located in Leiden, the Netherlands) and the Central Committee on Research Involving Human Subjects (the Hague, the Netherlands) on November 28, 2019. All subjects gave written informed consent prior to enrollment in the trial.

#### Study design and population

We performed a single-center, randomized study on the effect of an opioid on ventilation as measured by the steady-state hypercapnic ventilatory response (HCVR). We studied 24 adult healthy individuals (12 men/12 women; mean age 23 years with range 19-33 years, body mass index 23.4 kg/m^2^ with range 19.1-28.7 kg/m^2^, and weight 74 kg with range 57-103 kg). A 20 G arterial line was inserted in the radial artery of the left or right arm for blood sampling. Subjects received a low (100 mg) or high oral dose (200 mg) of the opioid tapentadol immediate release (Grünenthal GmbH, Aachen, Germany). Each subject only received one dose, and the order of study drug allocation was random and double blind. The drug was ingested with 100 mL tap water after a control HCVR was obtained. Thereafter HCVRs were obtained at 1-h intervals for 8 hours.

#### Study endpoints/outcome measures

To measure ventilation (${\dot{V}}_{E}$), the participants inhaled a gas mixture *via* a face mask coupled to a pneumotachograph and pressure transducer system (Hans Rudolph Inc., USA). The gas mixture consisted of O_2_, CO_2_ and N_2_ delivered by three mass flow controllers (Bronkhorst BV, the Netherlands) that were controlled by custom-made software (Leiden University). The software allowed the collection of ventilation data on a breath-to-breath basis and enabled variations in inspired gas concentrations to achieve the desired end-tidal oxygen and carbon dioxide concentrations (P_ET_O_2_ and P_ET_CO_2_), independent of the ventilatory response. The concept of this dynamic end-tidal forcing approach is described elsewhere in detail.^22^^,^^23^ In brief, in this study, subjects initially inhaled a normoxic gas mixture (P_ET_O_2_ 110 mmHg) without any added inspired CO_2_ for 5-6 min, after which we applied three to four steps in P_ET_CO_2_ with a fixed sequence: 4.5, 9, 13.5 and 18 mmHg above resting P_ET_CO_2._ In case the procedure tended to last longer than 20 min for the first three CO_2_ steps, the last step (18 mmHg) was omitted. The duration of each step was 6-8 min and ended when we observed a steady state in ${\dot{V}}_{E}$ (at least 2 minutes no further increase in ${\dot{V}}_{E}$). In-between HCVRs the subjects breathed room air. Inspired and expired O_2_ and CO_2_ were measured at the mouth (Datex Capnomac, Finland), and for monitoring purposes the ECG and oxygen saturation (Datex Cardiocap, Finland) were measured throughout the experiment day.

Blood samples were drawn from the arterial line for measurement of the tapentadol plasma concentrations. A 4 mL blood sample was drawn before dosing and at 1-h intervals for 7-h. The samples were analyzed by Ardena Bioanalytical Laboratory (Netherlands). Tapentadol concentrations were measured by tandem mass spectrometry (LC-MS/MS) methods, validated over a range of 0.1 to 1,900 ng/mL, in one batch. Within-run precision (% coefficient of variation) and accuracy (% bias) was 4.0 and 0.7 at the lowest level of quantitation, respectively, and 0.6 and 2.8 and the highest level, respectively.

### Method details

#### Sample collection

A formal power analysis was not performed but we enrolled a convenience sample of 24 subjects with 12 subjects per dose, which is common practice in similar PK/PD studies.^24^ Subjects were randomized to a low or a high dose regimen using computer-coded randomization list by an independent statistician. which was made available to the pharmacy who prepared the drug in unmarked syringes.

### Quantification and statistical analysis

We performed a physiology-based pharmacokinetic/pharmacodynamic (PK/PD) analysis to determine the effect of the opioid on the different components of the HCVR: resting ventilation, resting P_ET_CO_2_, slope of the HCVR and ${\dot{V}}_{E}$ at an extrapolated P_ET_CO_2_ of 55 mmHg (${\dot{V}}_{E}$55). The pharmacokinetics and pharmacodynamics of were analyzed with NONMEM VII (Icon Plc., USA), a software package for nonlinear mixed effects modeling, using a population approach.^25^

The pharmacokinetic data were analyzed first, using a two-compartmental model. Parameter estimation was done using Stochastic Approximation Expectation Maximization (SAEM), followed by objective function evaluation using Importance sampling (IMP). Rather than using an absorption compartment, we used the zero-order infusion duration from tablet into the blood. The adequacy of the pharmacokinetic model was evaluated by inspection of the individual data fits and the goodness-of-fit plots: measured concentrations *versus* individual predicted concentrations, measured concentrations *versus* population predicted concentrations, conditional weighted residuals *versus* time, and normalized prediction discrepancy error *versus* time. No covariates were included in the pharmacokinetic model analysis. The output of the PK analysis was used as one of the input variables to the pharmacodynamic analysis.

The ${\dot{V}}_{E}$ data were averaged over 1-min and these 1-min averages were used in the data analysis. The complete ${\dot{V}}_{E}$ dataset obtained during one HCVR run was analyzed to obtain the HCVR curve (Figure 1). To that end, we defined various components of the response that were included in the analysis: resting ventilation (${\dot{V}}_{B}$), resting P_ET_CO_2_, the ventilatory recruitment threshold (VRT) or the P_ET_CO_2_ at which ventilation starts to rise linearly, and the slope of the HCVR (S). Resting data were obtained prior to any CO_2_ inhalation. We also calculated for each HCVR curve the extrapolated ${\dot{V}}_{E}$ at a P_ET_CO_2_ of 55 mmHg (${\dot{V}}_{E}$55), as an additional measure of drug effect. Finally, we quantified the hysteresis between plasma opioid concentration and effect (blood-brain equilibration half-life or h½) and opioid potency (C_50_ or the opioid concentration in the brain causing a 50% effect); h½ and C_50_ were determined for all parameters, ${\dot{V}}_{B}$, resting P_ET_CO_2_, VRT, S and ${\dot{V}}_{E}$55 independently. By including the hysteresis in our models, the HCVR becomes a function of the effect-site PCO_2_ or PCO_2_ at the site were CO_2_ is sensed.

The relationship between CO_2_ content (C) and its partial pressure (P) was assumed to be linear, so that P = λ_0_ × C, where λ_0_ = 0.115 mmHg/(mL CO_2_ in 100 mL blood). The following CO_2_ alveolar mass balance equations were used for the lungs and body (approximating the body by one compartment):^8^^,^^21^

$$V_{ALV}\cdot \frac{d\left({arterial\;P}_{CO_{2}}\right)}{dt}=\left({\dot{V}}_{E}-{\dot{V}}_{D}\right)\cdot \left({inspired\;P}_{CO_{2}}-{arterial\;P}_{CO_{2}}\right)+\lambda_{1}\cdot \;\dot{Q}\cdot \;\left({venous\;P}_{CO_{2}}-{arterial\;P}_{CO_{2}}\right)$$

and

$$V_{TS}\cdot \frac{d\left({venous\;P}_{CO_{2}}\right)}{dt}=\dot{Q}\cdot \;\left({venous\;P}_{CO_{2}}-{arterial\;P}_{CO_{2}}\right)+\lambda_{2}\cdot \;{\dot{V}}_{CO_{2}}\text{,}$$

Where *V*_*ALV*_ is the alveolar volume, the arterial carbon dioxide pressure is assumed to equal alveolar pressure, ${\dot{V}}_{D}$ dead space ventilation, $\dot{Q}$ cardiac output, ${vP}_{CO_{2}}$ the venous carbon dioxide partial pressure, $V_{TS}$ the apparent CO_2_ distribution volume in tissue, ${\dot{V}}_{CO_{2}}$ the carbon dioxide production, λ_1_ = q × P_BW_/λ_0_/100 ≈ 10 and λ_2_ = 100 × λ_0_, where q = the volume conversion factor from standard temperature and pressure, dry to body temperature, and air saturated with water, and P_BW_ is the barometric pressure minus the pressure of air saturated with water. ${\dot{V}}_{ALV}$ was fixed to 3 L and ${\dot{V}}_{D}$ to 1.8 L/min; $V_{TS}$ and $\dot{Q}$ were estimated to be 10 and 4 L, respectively.

${\dot{V}}_{E}$ was assumed to depend on brain tissue carbon dioxide pressure (${brain\;tissue\;P}_{CO_{2}}$) as:

$$\tau \;\cdot \frac{d\;\left(brain\;tissue\;P_{CO_{2}}\right)}{dt}={arterial\;P}_{CO_{2}}-{brain\;tissue\;P}_{CO_{2}}$$

$${\dot{V}}_{E}=b{aseline\;\dot{V}}_{E}+S\;\cdot H\cdot \left(brain\;tissue\;P_{CO_{2}}-VRT\right)$$

and

$$H\left(x\right)=\delta \;\cdot \log\left[1+\exp\;\left(\frac{x}{\delta }\right)\right]$$

where *τ* is a time constant (estimated to be 2.5 min)^22^^,^^23^^,^^26^ and *H* is the so-called “hinge” function (Columbia University, a continuous hinge function for statistical modeling: https://statmodeling.stat.columbia.edu/2017/05/19/continuous-hinge-function-bayesian-modeling/), with δ fixed to 0.1 and *x* = (brain tissue PCO_2_ – VRT). For each respiratory run, parameters resting ${\dot{V}}_{E}$, resting P_ET_CO2, VRT, S were estimated or calculated (${\dot{V}}_{E}$55), which were then used to estimate h½ and C_50_ using sigmoid E_MAX_ models. The sigmoid E_MAX_ model had the form: $Effect\left(t\right)=\left(Effect\;at\;baseline\right)\times {\left[Parameter\;value\left(t\right)/Parameter\;value\left(t\right)+C_{50}\right]}^{\gamma }$ where γ is a shape parameter, where Effect at baseline is the control response prior to drug intake. P-values <0.01 were considered significant.

### Additional resources

Registration of the study was at the Dutch Clinical Trial Register, available at https://clinicaltrialregister.nl/en/trial/23681 (id 23681) on December 31, 2018.

## Data Availability

• All data reported in this paper will be shared by the lead contact upon reasonable request. • The NONMEM code reported in this paper will be shared by the lead contact upon reasonable request. • Any additional information required to reanalyze the data reported in this paper is available from the lead contact upon reasonable request.
